# The Impact of Natural Product Dietary Supplements on Patients with Gout: A Systematic Review and Meta-Analysis of Randomized Controlled Trials

**DOI:** 10.1155/2020/7976130

**Published:** 2020-01-23

**Authors:** Juan Yang, Guangxi Li, Donglin Xiong, Tony Y. Chon, Brent A. Bauer

**Affiliations:** ^1^Division of General Internal Medicine, Mayo Clinic, Rochester, MN 55905, USA; ^2^Department of Pain Medicine, Shenzhen Nanshan People's Hospital, Guangdong Medical University, Shenzhen, Guangdong 518052, China; ^3^Guang'anmen Hospital, China Academy of Chinese Medical Sciences, Beijing 100053, China; ^4^Division of Pulmonary and Critical Care Medicine, Mayo Epidemiology and Translational Research in Intensive Care, Rochester, MN 55905, USA

## Abstract

Natural product dietary supplements (NPDS) are frequently used for the treatment of gout, but reliable efficacy and safety data are generally lacking or not well organized to guide clinical decision making. This review aims to explore the impacts of NPDS for patients with gout. An electronic literature search was conducted to retrieve data published in English language from databases from inception to August 14, 2019. Randomized controlled trials (RCTs) that compared NPDS with or without placebo, diet modification, conventional pharmaceutics, or the other Chinese medicine treatment for gout patients were included. Two authors screened the articles, extracted the data, and assessed the risk of bias of each included trial independently. Meta-analysis was performed using Review Manager version 5.3.5. *Results*. Nine RCTS were enrolled in this review. The methodological quality of the nine RCTs was poor. The study results showed that in the majority of trials, NPDS demonstrated some degree of therapeutic efficacy for joint swelling, pain, and activity limitation. In contradistinction, serum uric acid (SUA) level (SMD −1.80, 95% CI: −4.45 to 0.86) (*p* > 0.05) and CRP levels (*N* = 232; SMD, −0.26; 95% CI, −0.55 to 0.04) (*p* > 0.05) did not improve significantly. The incidence of adverse events (AEs) was not lower in the participants treated with NPDS (*N* = 750; RR, 0.47; 95% CI, 0.20–1.11) (*p* > 0.05). *Conclusion*. Current existing evidence is not sufficient to provide clinical guidance regarding the efficacy and safety of NPDS as a treatment for gout due to poor trial quality and lack of standardized evaluation criteria. Larger and more rigorously designed RCTs are needed in the future.

## 1. Introduction

Gout is a form of inflammatory arthritis resulting from the deposition of monosodium urate crystals in the synovial joints and soft tissues in the setting of hyperuricemia, i.e., elevated serum uric acid level (SUA >6.8 mg/dL) [1]. It is a chronic and often debilitating disease featured by recurrent swelling, redness and pain in one or multiple joints, which, if untreated or poorly controlled, can lead to limited musculoskeletal function, work-related disability, and significant morbidities such as hypertension, cardiovascular disease, chronic kidney disease, and poor health-related quality of life [2, 3]. The prevalence of gout, as well as its social and economic burden, has been rising globally, especially for middle-aged and elderly men. In 2007-2008, the prevalence of gout among US adults (8.3 million individuals) was 3.9% [4], and the incidence has continued to rise, due in part to economic development, dietary habit changes, aging population, *l*, and increasing-associated comorbidities [1, 5].

Both American and European rheumatology society guidelines for gout management focus on controlling gout flares and long-term reduction in total uric acid levels [6, 7]. Conventional medicines are limited in patients with gout because of interactions, inherent drug toxicity, and polypharmacy for patients with multiple comorbidities. Dietary therapy can lower uric acid levels, delay gout complications, and, in many cases, reduce or elimilate the need for conventional medications. Strong evidence suggests that diet is the most modifiable factor in gout management [8–10].

Natural product dietary supplements (NPDS), also named natural dietary supplements (NPS), are defined as systemically ingested, nonmineral, nonvitamin, and natural product-derived substances [11]. Previous studies have suggested that natural products including vegetables, nuts, legumes, fruits, and whole grains play an important role in the development of hyperuricemia and gout [12]. To date, no specific guidelines have recommended NPDS for gouty arthritis. Many publications have discussed the importance of natural compounds derived from animals, plants, and microbe sources for the treatment of human diseases [13]. Natural products are often regarded by consumers and patients as gentle and safe to human body because of their natural properties. In reality, the active ingredients of these natural products are compounds, which, if powerful enough to provide a beneficial effect, may also be powerful enough to cause adverse effects [14]. Natural products can regulate both the production and the excretion of uric acid, which may benefit the treatments of hyperuricemia, as reported in a previous review [15]. Several herbal compounds have been found to have antihyperuricemic effects in vitro and in vivo, suggesting they may play a role in the treatment of gout arthritis, while further research studies are needed to explore their potential action [16].

Standard epidemiology and treatment are required to further evaluate and verify the clinical efficacy and safety of the NPDS in gout treatment; therefore, providing valid and reliable data for accurate application of NPDS therapy are needed in the future [17].

Hence, our review aims to reveal some insights from previously published, randomized, controlled trials (RCTs) exploring the impacts of NPDS on gout patients and presents the evidence for natural dietary.

## 2. Materials and Methods

### 2.1. Search Strategy

**Figure 1:**
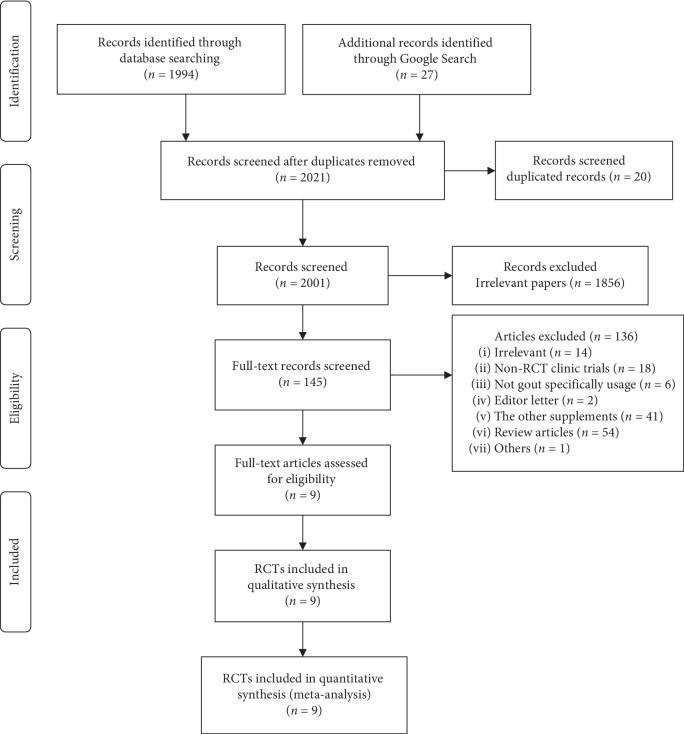
PRISMA flow chart.

A comprehensive electronic literature search was performed in the following databases from the database inception to August 14, 2019, limited to English language only, and excluding animal studies: Ovid MEDLINE (R) and Epub Ahead of Print, In-Process & Other Non-Indexed Citations and Daily, Ovid Cochrane Central Register of Controlled Trials, Ovid Embase, Ovid Cochrane Database of Systematic Reviews, and Scopus. Search terms used were (natural product OR diet OR supplement OR remedy OR remedies OR medicine OR prescriptions OR preparations OR extract OR indigenous OR traditional OR alternative OR complementary OR primitive OR Chinese OR China OR Japan OR Kampo OR oriental OR Asian OR Korean OR native American OR Indian OR Hindu OR siddha OR Tibet OR Africa OR Brazil OR rongoa OR Ayurveda OR herb OR natural OR plant OR flower OR fruit OR leaf OR leaves OR tea) AND (antigout OR anti-gout OR gout OR gouty OR hyperuricemia). All retrieved papers were manually scanned to identify further possible articles missed by electronic searching. Two reviewers screened the publications independently; any discrepancy between the two reviewers was resolved by consensus or by a discussion with a third reviewer if needed. The quality of RCTs was assessed using the Cochrane Collaboration Risk of Bias Assessment Tool [18]. The PRISMA flow chart is shown in Figure 1.

### 2.2. Participants

All subjects were aged 18 years or above diagnosed with gout based on the diagnostic criteria [6].

### 2.3. Interventions

All trials evaluating any natural product or natural compounds derived from animals, plants and microbe sources alone or in combination were included.

### 2.4. Comparators

All trials with any control groups such as no treatment/waiting list, sham therapy/placebo, nonpharmacological therapy (e.g., diet modification), and pharmacological therapy (e.g., allopurinol), alone or in combined therapies, were enrolled.

### 2.5. Outcomes

Outcome measures should include at least one or more of the following measurements: (1) pain relief evaluated by the visual analogue scale (VAS) or numeric rating scale (NRS); (2) joint function improvement by the NRS; (3) health-related quality of life by 36-Item Short-Form Health Survey (SF-36) and the Gout Assessment Questionnaire; (4) clinical efficacy identified by reduction in SUA levels; (5) inflammation markers by white blood cell (WBC) count, erythrocyte sedimentation rate (ESR), and C-reactive protein (CRP) level; and (6) safety was monitored by the reporting of adverse events (AEs).

### 2.6. Selected Trials

RCTs involving human participants were included in this review. Any study focused on the efficacy and safety of NPDS (single or compound) on gout was included. The abstract publication languages were restricted to English language only.

### 2.7. Data Extraction

Two review authors extracted relevant information independently from the retrieved trials. An Excel spreadsheet was designed to record descriptive data, the methodological quality of original studies, treatment regimen and duration, outcomes, efficacy, and AEs. The raw data such as means and standard deviations (SD) for continuous outcomes and event numbers or participants for dichotomous outcomes were extracted. Any disagreement between the two reviewers was resolved by a discussion between the two reviewers or referring to the original author.

### 2.8. Assessment of Risk of Bias in Included Studies

The potential bias of each trial was assessed using Cochrane Risk of Bias Tool for Randomized Controlled Trials [19] by two reviewers independently in the following domains: risk of selection, reporting, performance, detection, attrition, and other sources of. Each domain was assessed as either “high,” “low,” or “unclear” bias. Any discrepancy was resolved by consensus or resort to a third review author.

### 2.9. Data Synthesis

Treatment efficacy was analyzed with the Cochrane Collaboration's statistical software, RevMan version 5.3.5 (Cochrane Collaboration, London, UK). Outcomes of continuous variables were presented as mean differences (MDs) between the observation and control groups with the corresponding 95% confidence intervals (CIs). A meta-analysis was performed only if the studies were sufficiently homogeneous. Results of dichotomous data were presented as risk ratios (RRs) with the corresponding 95% CIs. The random-effect model was applied, if *I*^2^ >50% indicated significant heterogeneity among the studies. Subgroup analysis was performed according to the different NPDS interventions. The study number of each outcome was insufficient, so publication bias could not be analyzed with a funnel plot.

## 3. Results

### 3.1. Characteristics of the Trials

**Figure 2:**
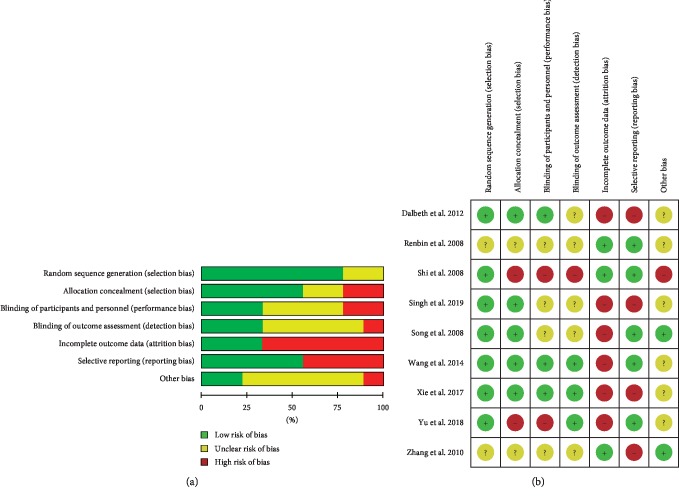
Risk of bias summary.

The literature search retrieved 2021 references, of which 20 were duplicates; 1856 articles were rejected on reading their titles and abstracts, and 145 articles were identified for detailed review. Finally, 9 articles were included (Figure 2).

A total of nine RCTs with 1156 participants were included in this review. Seven trials were conducted in China [20–26], 1 in the USA [27], and 1 in New Zealand [28]. All the studies were published from 2008 to 2019. Seven studies evaluated herbal therapies, with the remaining two evaluating skim milk and cherry extract. Six trials had two arms; two trials had three arms; one trial had four arms. Five trials reported natural product formulation in decoction, two in capsules, and one in powder. Five trials recorded gout flare-up; four trials did not record gout flare-up. Five trials reported no AEs; four trials reported AEs. Two trials assessed the compliance of patients (Table 1).

### 3.2. Methodological Quality

Seven of the nine RCTs had lower risk regarding randomization due to reported methods of sequence generation. Three trials used a random number table [22, 24, 28], two trials referred to SAS software for setting the random variable seeds [20, 25], one adopted the random envelope method [21], and the other used online computer random number generator [27]. The other two trials had an unclear risk for random sequence generation due to lack of detail [23, 26]. Two trials had high-risk bias for allocation concealment [20, 25]. Two trials had unclear randomization without description in sufficient detail [23, 26]. Five trials had lower risk bias for allocation concealment [21, 22, 24, 27, 28]. Three trials were double-blinded trials with a lower risk for blinding participants and study personnel [21, 22, 28]. Two trials did not use a blinding method to participants and study personnel during the study with a high risk due to the great appearance difference between Chinese medical formulation pills and decoction with conventional pharmaceutics such as indomethacin, benzbromarone, and allopurinol [20, 25]. The other four trials had insufficient information to permit a judgment [23, 24, 26, 27]. Three trials could be regarded as lower risk for blinding of outcome assessors [20–22]. One trial had high risk in outcome assessment due to a lack of blinding [25]. Five trials had unclear risk in outcome assessment without description in sufficient detail [23, 24, 26–28]. Two trials reported cases with poor compliance which were dropped from the study [22, 24], two trials reported cases lost to follow-up [21, 28], and two trials had missing values in the results [20, 27], which might affect the results. Four trials were considered as a high risk of attrition bias with selective data reporting [21, 23, 27, 28]. The other five trials were classified as low risk [20, 22, 24–26]. For poor blinding and allocation design, one trial was assessed as a high risk of bias in the other resource [25]. Two trials were estimated at a lower risk because of their study design [23, 24]. The other six were regarded as an unclear risk of other sources of bias [20–22, 26–28]. The methodological quality of the nine RCTs was poor because of two or more criteria listed as a high or unclear risk of bias. The study methodology was summarized in Figure 2.

### 3.3. Measurable Outcomes

#### 3.3.1. Joint Swelling, Pain, and Limitation Relief

Study results suggested that NPDS treatments were superior to control interventions in joint swelling, pain, and activity limitation, except one trial which reported improvement just in joint pain-relieving, not in swelling and activity limitation [22]. In the double-blind RCT, 176 participants with acute gouty arthritis were divided to either the chuanhu antigout mixture 250 ml orally daily plus placebo (mimetic agent of colchicine) or colchicine 1 pill (0.5 mg/pill) orally, twice daily, for 3 days and later once daily. All the participants orally administered pain-reliving medication etoricoxib 60 mg once for 10 days. Results indicated that the chuanhu antigout mixture had a favorable effect in decreasing of joint pain score, instead of joint swelling and limitation compared with colchicine [22].

The evaluation criteria for therapeutic effect of joint swelling, pain, and activity limitation varied in the enrolled trials. Besides three trials [21, 22, 27], the other six trials have different criteria to assess the therapeutic effect [20, 23–26, 28]. To investigate the compatibility of modified prescriptions of simiao pill on patients with acute gouty arthritis, Shi et al. [25] evaluated the clinical efficacy with “guiding principles of clinical research on new drugs of traditional Chinese medicine,” which included 4 grades (none, mild, moderate, and severe), with scores 0, 1, 2, and 3, respectively. Affected joint arthralgia, erythema, and swelling, as well as blood uric acid level and gout recurrence were evaluated comprehensively. In the other trial to observe the efficacy of the modified simiao tang for gouty arthritis and blood uric acid, Renbin et al. [26] used “an assemblage of guiding principles of clinical and preclinical research on new drugs (western drugs),” which included “clinical cure, markedly improved, improved, and ineffective.” The index of swelling and pain in the joints were scored according to the swelling and pain in the joints before and after treatment. Song et al. [24] classified the therapeutic effect in three grades: “markedly effective, effective, and ineffective” on the basis of the criteria “diagnosis and curative effect standards of traditional Chinese medicine disease.” These criteria covered joint symptoms and signs as well as laboratory indices. Zhang et al. [23] used the criteria to assess efficacy effects with three categories of “cured, improved, and failed,” based on both joint swelling and pain. The affected joint pain was assessed with the assessment of the Budzyuski index, which graded in 0, 1, 2, 3, 4, and 5 with increasing pain severity. Dalbeth et al. [28] recorded gout flare and severity of pain with the 10-point Likert scale. Yu et al. [20] adopted the SF-36 scale to evaluate the change in affected joint swelling, pain and activity. The levels of SUA and urine urate were measured at week 0 and week 4. A meta-analysis of joint swelling, pain, and limitation relief could not be employed due to the inconsistency and heterogeneity of interventions, controls, and outcome measures.

#### 3.3.2. Serum Uric Acid (SUA) Level

**Figure 3:**
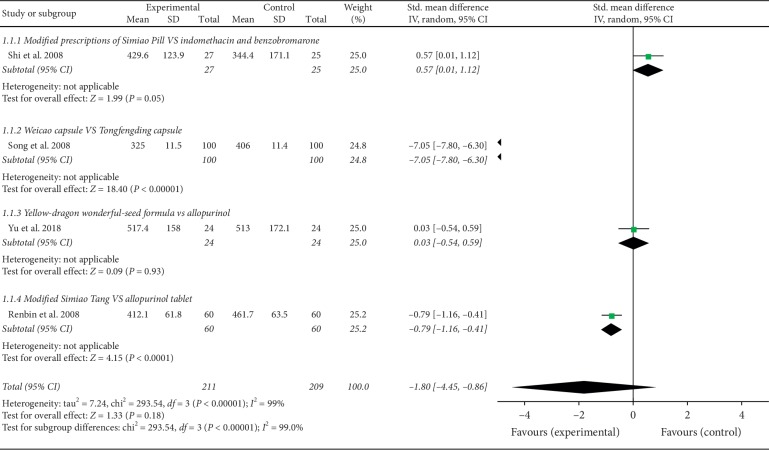
Effects of NPDS on the serum uric acid (SUA) levels of patients with gout.

Four RCTs included 420 patients (211 patients in the observation groups and 209 in the control groups) provided SUA changes before and after treatment. The study result showed that there was no significant difference between the NPDS interventions and the control groups in terms of SUA reduction (SMD −1.80, 95% CI: −4.45 to 0.86) (*p* > 0.05). Because *I*^2^ = 99%, a random-effect model was used for the analysis. Subgroups were divided by treatment; the subgroup meta-analysis showed that modified simiao tang was better in reducing SUA than the allopurinol tablet [26]. Due to the lack of detail in the reports, no meta-analysis was conducted in one-third of the trials [21–23, 28]. In addition, the other study reported serum urate value instead of SUA level [27] and was temporarily omitted from the meta-analysis. The effects of NPDS on the serum uric acid (SUA) levels of patients with gout are shown in Figure 3.

#### 3.3.3. CRP Value

**Figure 4:**
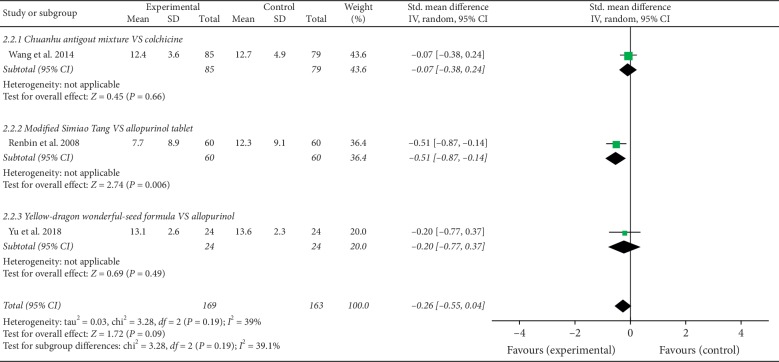
Effects of NPDS therapies on the CRP levels of patients with gout.

Four studies [20, 22, 26, 28] reported CRP value changes pre- and posttreatment, and the meta-analysis of three trials [20, 22, 26] showed that the NPDS therapies were not more effective than treatment with conventional pharmaceutics such as allopurinol and colchicine (*N* = 232; SMD, −0.26; 95% CI, −0.55 to 0.04) (*p* > 0.05). In the subgroup meta-analysis, modified simiao tang was superior to allopurinol tablets in decreasing CRP level [26]. The other study [28] which reported CRP change roughly was just temporarily omitted from the meta-analysis. The effects of NPDS therapies on CRP levels of patients with gout are shown in Figure 4.

#### 3.3.4. Adverse Events

**Figure 5:**
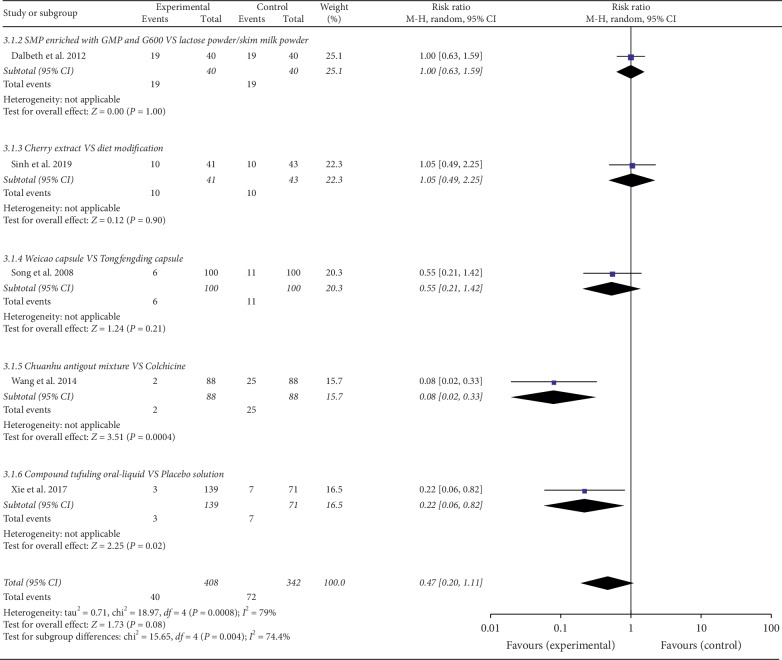
Adverse events caused by NPDS interventions in patients with gout.

Five RCTs including 750 patients with 408 in the experimental groups and 342 in the control groups provided safety evaluation data [21, 22, 24, 27, 28]. The meta-analysis showed that NPDS therapies had not fewer adverse events than the control groups (*N* = 750; RR, 0.47; 95% CI, 0.20–1.11) (*p* > 0.05). In total, 112 patients experienced side effects. In the subgroup meta-analysis, chuanhu antigout mixture and compound tufuling oral-liquid had fewer AEs than colchicine and placebo solution separately [21, 22] (*p* < 0.05). The AEs included bitter taste, poor appetite, and leucopenia, as well as gastrointestinal reactions such as nausea, vomiting, diarrhea, and flatulence, which could be relieved by temporary dosage reducing or medication pause [22, 24, 28]. Xie and his colleagues [21] found fewer leucopenia incidence (2.16%) in the treatment group of the compound tufuling oral liquid compared with that of the placebo solution (9.86%), and more detailed information was not further reported. No other serious adverse events were recorded. Adverse events caused by NPDS interventions in patients with gout are shown in Figure 5.

## 4. Discussion

Nine RCTs were included in our review, which compared Chinese herbs, cherry extract, and compound skim milk powder with a placebo control, conventional pharmaceutics, diet modification, and other Chinese medications for the treatment of gouty arthritis. Outcome measurements cover affected joint swelling, pain, and activity relieving, SUA and CRP value change, and AEs incidence.

The methodological quality of the included trials was poor with two or more assessments of high or unclear risk of bias. Despite mostly positive results in the therapeutic efficacy evaluation, the evidence data evaluating clinical efficacy were not convincing and robust, which could be attributed to the variety of evaluation criteria. Some studies comprehensively evaluated joint arthralgia, swelling, and activity limitation as well as laboratory indices [24, 25], some focused on joint swelling and pain [23, 26, 28], and one utilized SF-36, SUA and urine urate for assessment [20]. Poor methodological quality and the small number of the included trials were the other contributors. Therefore, to fully assess the clinical role of NPDS in the treatment of gout, high-quality RCTs of standardized evaluation criteria with international recognition are required in the future.

Until now, nonsteroidal anti-inflammatory drugs (NSAIDs), colchicine, corticosteroids, and prophylaxis are guideline-recommended pharmaceuticals with high-strength evidence for individuals with gout [2], but the use of these drugs is often associated with unwanted side effects and adverse events [29]. NPDS interventions attract a great deal of public attention due to their perception as being nontoxic, natural, and economical properties [16]. Our review suggests that NPDS interventions may have some clinical efficacy in terms of symptom improvement, reduction in SUA and CRP levels, all with a lower level of adverse events compared with standard pharmacological treatment. They may possibly be associated with potential toxicity due to their multiherbal components and different formulations (e.g., decoctions and powders) with may be associated with gastrointestinal reactions.

Numerous articles have been written describing the important role and safety of NPDS therapies in patients with gout [15, 16, 30–34]; but until now, no review has quantitatively synthesized the relevant RCTs. To our knowledge, this review is the first overview of available RCTs investigating the efficacy and safety of NPDS approaches in gout management. This review highlights the impact of natural products on gout management and permits the identification of current evidence gaps, thus informing clinical decision making and guiding future research.

This review does have limitations. First, due to the study design limitation, we only searched RCTs published in English, thus possibly omitting important studies which appeared only in non-English journals. In particular, Chinese language journals may be an additional resource worthy of systematic review. Second, the included RCTs are of low quality. This makes the comparative analysis difficult to perform and reduces the confidence in the meta-analysis. Finally, the heterogeneity of the included studies was significant. More high-quality trials with large sample size RCTs are required in the future.

## 5. Conclusion

This review provides insight into the contemporary treatment of gout with NPDS. NPDS appeared to be superior to control groups in affected joint pain, swelling, and activity limitation, while not in decreasing SUA and CRP levels or the incidence of AEs. Current existing evidence is insufficient to permit a definitive statement about the efficacy and safety for gout patients due to poor trial quality and standardized evaluation criteria. Further larger and more rigorously designed RCTs are needed in the future.

## Figures and Tables

**Table 1:** Characteristics of the included studies.

Author	Year	Country	Condition	Arms	Population	NPDS	Formulation	Control group	Evaluation criteria	Therapeutic efficacy
Shi et al. [25]	2008	China	Gout	4	107	Modified prescriptions of simiao pill	Pill	Indomethacin and benzbromarone	Guiding principle of clinical research on new drugs of traditional Chinese medicine	Joint arthralgia, erythema, and swelling, blood uric acid and gout recurrence↓
Renbin et al. [26]	2008	China	Gouty arthritis	2	120	Modified simiao tang	Decoction	Allopurinol tablet	An assemblage of guiding principles of clinical and preclinical research on new drugs	The index of swelling and pain in the joints, BUA, and CRP ↓
Song et al. [24]	2008	China	Gout	2	200	Weicao capsule	Capsule	Tong Feng Ding capsule	Diagnosis and curative effect standard of traditional Chinese medicine disease	Joint swelling and pain, laboratory indices↓
Zhang et al. [23]	2010	China	Acute gouty arthritis	2	67	Blood-letting cupping plus herbal medicine	Decoction	Diclofenac sodium enteric-coated tablets	The Budzyuski index of pain	Joint pain and swelling, BUA↓
Dalbeth et al. [28]	2012	New Zealand	Gout	3	120	SMP/GMP/*G*600	Powder	GMP, G600	The 10-point Likert scale	Gout flare, the severity of joint pain↓
Wang et al. [22]	2014	China	Acute gouty arthritis	2	176	Chuanhu antigout mixture	Decoction	Colchicine	—	Gout recurrence, CRP, AEs↓
Xie et al. [21]	2017	China	Intercritical and chronic gout	2	210	Compound tufuling oral liquid	Decoction	Placebo solution	—	The frequency of recurrent joint swelling and pain, BUA↓
Yu et al. [20]	2018	China	Gout with hyperuricemia	3	72	Yellow-dragon Wonderful-seed Formula	Decoction	Allopurinol group	SF-36	Joint pain, BUA, and CRP↓
Singh et al. [27]	2019	USA	Gout	2	84	Cherry extract	Capsule	Diet modification	—	Joint pain intensity↓, average pain in last 24 h↓

NPDS: natural product dietary supplement; CRP: C-reactive protein; BUA: blood uric acid; SMP: skim milk powder; GMP: glycomacropeptide; G600: G600 milk fat extract.
